# Parliament and the Profession

**Published:** 1916-02-26

**Authors:** 


### PARLIAMENT AND THE PROFESSION.

### The Business of the Session.

THE nature of the business that will be transacted during the session of Parliament which began on February 15 was defined in the following passage of the King's Speech: "The only measures which will be submitted to you are such as in the opinion of My advisers tend to the attainment of our common object." That is to say, that any Government proposals that may be brought forward will be designed for the purpose of the successful prosecution of the war. This programme, however, does not rule out measures affecting the medical profession, and evidence is to be found in the debate on the Address and in questions already addressed to Ministers that the House of Commons fully appreciates the important part which the profession is playing in the present crisis.

### Sale of Petrol to Doctors.

REPLYING to the Earl of Ronaldshay, the Prime Minister denied that the Government had imposed any restrictions upon the sale of petrol, but he stated that certain firms might find it necessary to curtail their supplies to other customers. Should this be the case, it is obviously desirable that steps should be taken to ensure that doctors with motor-cars have the first call on such supplies as are available after the Government requirements are satisfied.

### War-Time Charities.

IN THE HOSPITAL of January 22 (p. 371) it was suggested that it should be the business of some duly-constituted authority to control the activities of war-time charities. This question was raised in the House of Commons on February 17, when Mr. Anderson asked the Prime Minister whether the Government proposed to take any steps in respect of war charities generally, with a view to the protection of donors and all urgent *bona-fide* charities. On behalf of the Prime Minister, Mr. Long stated that arrangements had been made for giving official recognition to appeals on behalf of the Belgians which are approved by the British and Belgian Governments, but it seemed to be an almost impossible task to extend this principle to war charities generally.

### Drugging Sailors and Soldiers.

THE question of the administration of narcotic drugs to sailors and soldiers was raised by Mr. Gilbert, who asked the Home Secretary whether he had had any reports from the police showing that a number of men had been drugged in a certain district in South London. Mr. Samuel replied that no evidence had been forthcoming inconsistent with the view that the symptoms were produced by the drinking of strong spirits. He added that the administering of drugs is a criminal offence, but that no single case had been substantiated on investigation. This is quite apart from the question of the sale of cocaine and morphine to soldiers referred to in THE HOSPITAL last week (p. 446), but there is reason to believe that the Government is prepared to take very strong action should it be found that this practice is persisted in.

### Armlets for the Unfit.

ACCORDING to Mr. Thomas, rejected recruits are experiencing considerable difficulty in securing an armlet unless they submit to a further medical examination. Mr. Tennant explained, however, that men who are able to produce forms showing the date and cause of rejection are not required to undergo further medical examination, but if the cause of rejection is not stated on the certificates, it is necessary for the man to be re-examined.

### Diseases Contracted during Service.

ON the second day of the session Mr. Thorne opened a debate on the question of the payment of pensions and allowances to all sailors and soldiers discharged from the Navy and Army on account of diseases contracted or developed during service with the Colours. His case was that every sailor or soldier who is turned away as medically unfit ought to receive the same amount of pension as a man who had been to the Front and been discharged in consequence of wounds. In the course of a prolonged discussion—in which, by the way, there were several criticisms of the decisions of the medical board—the Financial Secretary to the War Office stated the position of the Government in the matter. A man, he said, who contracts some disease and is invalided out of the Service is examined by a medical board, who certifies either that the disease was due to his service or was not. If the man is certified by the medical board to be suffering from some disease which, though it may have occurred in, is not really due to his military service, he has, under the terms of the Royal Warrant, no claim to a pension. But it is quite a mistake to suppose that there is no appeal from the medical board. Mr. Forster added that he had known a great many cases in which an appeal had been made to the higher medical authorities at the War Office and to the Director-General, who brought to bear on the examination of these cases the most expert knowledge and great sympathy. In the end the amendment was by leave withdrawn, but the debate served the useful purpose of showing that the opinion of the House is strongly in favour of giving discharged soldiers the benefit of any doubt there may be as to whether the disease on account of which he was discharged was due to service with the Forces. The matter was again referred to on the following day, when questions adressed to Mr. Tennant elicited the information that out of 35,500 men discharged from the Army for disability up to the end of 1915, about 12,000 had received no pension. The 12,000 consists chiefly of men who upon their unit being ordered abroad were not found equal to the strain of active service in the field.

